# Use of a Handheld X-ray Fluorescence Analyser to Quantify Chloride Ions In Situ: A Case Study of Structural Repair

**DOI:** 10.3390/ma14030571

**Published:** 2021-01-26

**Authors:** Servando Chinchón-Payá, Julio E. Torres Martín, Nuria Rebolledo Ramos, Javier Sánchez Montero

**Affiliations:** Instituto Eduardo Torroja Ciencias de la Construcción (IETcc-CSIC), Calle de Serrano Galvache, 4, 28033 Madrid, Spain; juliotorres@ietcc.csic.es (J.E.T.M.); nuriare@ietcc.csic.es (N.R.R.); javier.sanchez@csic.es (J.S.M.)

**Keywords:** chloride ions, cements, reinforced concrete, X-ray fluorescence, handheld XRF analyser

## Abstract

To ensure that a structure will last throughout its service life, repairing reinforced concrete entails, among others, correctly marking off the area affected by aggressive agents that may deteriorate both the concrete and the steel. Chloride, the most damaging source of reinforcement corrosion, may diffuse to a greater or lesser distance from the surface depending on the ease of penetration. In this study, we calibrated a handheld X-ray fluorescence analyser (hXRF) and used it to quantify the chloride concentration in cement-based materials. The findings were verified against a series of samples of known concentration to establish a suitable correction factor. Chloride ions were quantified precisely and accurately with the hXRF instrument, and we calculated a correction factor of 1.16. The instrument and the information recorded were used to quantify the chloride ion content in different parts of an existing structure. The analyser identified apparently healthy areas that could, nonetheless, pose oxidation problems in the near future due to significant chloride concentration. Chloride quantification data at different depths can be used to draw diffusion or penetration profiles and to determine whether ion concentration around the reinforcement is within the recommended limits. The method developed can be applied in situ to quickly locate the most critical areas.

## 1. Introduction

A number of factors can shorten the service life of reinforced concrete structures due either to concrete deterioration or steel reinforcement corrosion, the latter predominantly induced by the presence of chloride ions.

Reinforced concrete is damaged by chloride ions due to their capacity to activate the passive layer that protects the steel, exposing the metal to oxidation [1,2,3]. Steel reinforcement oxidation may be attributed to flaws spanning the full spectrum from fissures in the concrete to the brittle failure of structural members [4,5,6,7,8].

Although many mechanisms may be involved in chloride ingress in concrete, diffusion is widely agreed to be the most prominent. Ingress and related service life models have been developed from empirical variations based on Fick’s laws [9,10].

The basis for normalising chloride quantification is the Volhard method [11], in which a silver salt is used to prompt the precipitation of chloride ions dissolved in a solid sample previously digested in an acid medium. On the basis of the chemical behavior between silver and chloride, sensors have been made to quantify chloride ions in concrete directly [12,13]. The performance of an embedded chloride sensor in cementitious materials depends on many factors, from concrete properties to sensor surface conditions [14] and some studies have reported good performances of such sensors [15,16]. However, some differences in the quantification of chloride ions may appear depending on the method used, because the sensors detect the ions in the interface sensor/cement paste while the destructive normalised methodology quantifies the total amount of chloride ions by the cement weight.

X-ray fluorescence (XRF) spectrometry involves measuring secondary or fluorescent energy emitted when a sample is radiated with high energy X-rays (wavelength from 0.01 to 10 nm). Secondary X-ray emission is characteristic of the atoms in a sample, making this a useful technique for the detection and quantification of elemental concentrations. The European standard [17] for the chemical analysis of cement includes a section endorsing XRF as an alternative method for quantifying the chemical components of a sample, providing it is not deemed to be a primary reference and the instrument is duly calibrated to suitable standards. Both methods for quantifying chloride ions entail gathering samples from the structure studied and forwarding them to a laboratory for testing, a time-consuming and costly procedure. Handheld X-ray fluorescence analysers (hXRFs) are less expensive than traditional XRF and offer an easy-to-use alternative, however they are subject to some limitations intrinsic to the analyser, including a high detection threshold that hinders the identification of low atomic weight elements and insufficient precision in elemental quantification across wide concentration ranges. Manufacturers have addressed those constraints by building a number of operating modes into such devices. The soil mode is optimal for trace and minor elements; the mining mode is useful for quantifying majority elements; the hybrid mode delivers semi-quantitative data in both scenarios [18]. Nonetheless, before using an hXRF analyser, it should be calibrated for the intended purpose to avoid the matrix effect.

The is a wide range of threshold values above which chloride ions are deemed to be detrimental to reinforcement, from [Cl^−^]/[OH^−^] ratios of 0.12 to 3.0 to free chloride content of 0.03–4% [1,19,20,21,22,23] by cement chemical composition, temperature, and reinforcement composition [3]. The wide range of values may be due to the concrete characteristics and the environmental conditions, but the composition of the steel and its finish may also have an effect [24,25,26]. The Spanish structural concrete code [27] defines the threshold above which steel may oxidise at 0.6% by cement weight. Eurocode 2 [28] also cites 0.6% by cement weight as the maximum for reinforced concrete and 0.3% by cement weight for prestressed concrete.

Techniques are now in place for the non-destructive, in situ determination of reinforcement corrosion current density in concrete structures [29]. Such techniques can be used to assess both the efficacy of repairs and the condition of existing structures. A combination of the two approaches can result in suitably conducted repairs and subsequent assessments.

In this paper, we describe the calibration and adaptation of a handheld X-ray fluorescence analyser to quantify chloride ions in mortar or concrete samples and its application to the in situ assessment of structures. The threshold chloride concentration cited in the existing legislation were taken as a criterion for marking off the areas affected by chloride ions. We conclude with the description of a case study in which the hXRF supported structural analysis and repairs by determining the chloride concentration at different points on a corroded structure.

## 2. Materials and Methods

The first stage of this study consisted of the laboratory calibration of an instrument to quantify chloride ions and, subsequently, validating the chloride concentration findings against the results of potentiometric titration of the samples [11]. Acid-soluble titration was conducted to quantify the total chloride ions in each sample. One potentiometric test was conducted and three hXRF readings were taken per sample, yielding a total of 91 samples.

Then, the calibrated instrument was used as a supporting tool to diagnose the causes of deterioration in a reinforced concrete structure and as an objective method for marking off the areas in need of rehabilitation and repair. The criterion applied was the 0.6% chloride content threshold set out in the applicable legislation, in this case the Spanish structural concrete code. The degree of corrosion in the reinforcing steel analysed was determined with a GECOR corrosimeter (James Instruments Inc., Chicago, IL, USA), an instrument that operates on the linear polarisation principle with current confinement to measure polarisation resistance (*R_p_*), i.e., by generating a 30 to 100 s galvanostatic pulse to induce quasi-stability. The reference for the corrosion current density readings delivered by the corrosimeter is the polarised rebar-bearing area within the sensor range. The *R_p_* was calculated directly as ΔE/ΔI multiplied by the rebar-bearing area, the technique most commonly used to measure corrosion current density in reinforcement steel. The GECOR’s Cu/CuSO_4_ sensors (James Instruments Inc., Chicago, IL, USA) afforded the instrument stability and ruggedness for field use [30]. 

Chloride concentration was found with a handheld Olympus Innov-X Delta XRF analyser (Olympus IMS, Waltham, MA, USA) [31] operating in soil mode and expressing the quantification findings in parts per million, equivalent in percentage to 1/10,000.

The test setup included a workbench on which the hXRF analysed the samples under radiation exposure-free conditions in which the readings could be taken without any actual hand contact with the instrument while in operation. The workbench was connected to a computer to monitor the tests. In on-site testing, both devices were powered by an external generator.

The concrete samples were removed from the structure analysed with a drill bearing a special concrete bit [32]. The dust generated during drilling was collected in a funnel-shaped plastic container to minimise material loss and subsequently decanted into cylindrical sample holders positioned on the workbench for analysis. Dust samples were carefully taken at different depths to determine the presence of chloride ions, and when affirmative, the extent of chloride ingress.

The field electrochemical readings taken with the GECOR device [29,33] included concrete resistivity, corrosion potential, and corrosion current density. The methodology involved mapping the corrosion potential and resistivity to identify the degree of corrosion by area [34,35]. As a parameter closely related to concrete moisture content, resistivity is useful for predicting corrosion risk. Here, it was found with the four-probe test (Wenner method [36]), in which four electrodes spaced at predefined distances were pressed onto the concrete surface. A current passed between the two outer probes and the voltage between the two inner probes was recorded. The area to be measured was premoistened and marked off with a cover meter to ensure readings were undisturbed by reinforcement steel proximity. The degree of corrosion risk by range of resistivity values is given in Table 1.

Then, corrosion current density, which is directly related to reinforcement section loss, was determined in the areas found to be most severely affected. The GECOR device measures the polarization resistance (*R_p_*) to calculate the corrosion current density according to the Stern–Geary equation (see Equation (1)) with a constant B value of 26 mV. The method used by the GECOR is the “modulated current confinement” where the sensor uses two concentric counter electrodes, the external one called the “guard ring” which confines the galvanostatic signal to a determined area [32,33]. The risk of corrosion as assessed on the grounds of *i_corr_* values is given in Table 2. The Stern–Greary equation is as follows:

(1) $$Icorr=\;\frac{B}{Rp}$$

Concrete can only be deemed to be fully corrosion risk free, where the corrosion current density for all the points measured is <0.1 µA/cm^2^.

### 2.1. Samples

A total of 91 mortar and concrete samples, which were prepared with ordinary Portland cement with an initially unknown chloride content, were analysed in the laboratory. Some of the samples were taken from cores that had been removed from existing structures and others from concretes and mortars prepared in the research group’s laboratory for prior chloride penetration studies. All the samples were also automatically titrated with end-point potentiometry [11].

### 2.2. On-Site Use of the hXRF

There were two objectives for the on-site use of the hXRF. On the one hand, it confirmed the pathology affecting the structure and, on the other hand, it was used to identify and mark the areas of the concrete in need of repair based on an objective criterion, in addition to only visual identification of the extent of deterioration.

**Figure 1:**
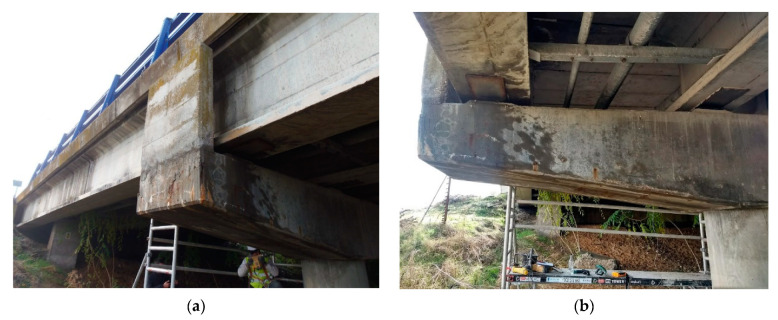

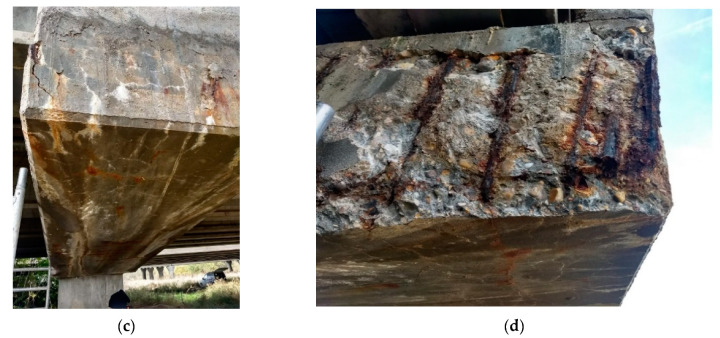
(**a**,**b**) Structure analysed from different perspectives; (**c**,**d**) Detailing (photos taken by S. Chinchón-Payá).

The concrete girder, depicted in Figure 1, which was the object of the case study, was characterised by a section that tapered outward from the centre of the viaduct to the edges. Substantial concrete deterioration was observed in areas exposed to rain or runoff water, including reinforcement oxidation with detachment of the concrete cover in some areas.

The surface where the electrochemical readings were taken and the concrete dust samples were collected was the surface facing the scaffolding, visible in Figure 1 (the rear side of the member shown in Figure 1b.

**Figure 2:**
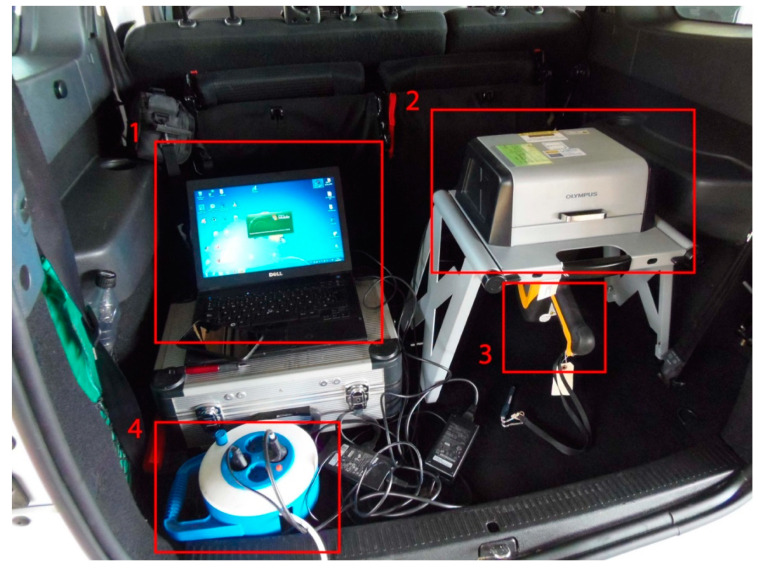
Experimental setup for in-situ chloride analysis. (**1**) Laptop computer; (**2**) Workbench; (**3**) Handheld X-ray fluorescence analyser (hXRF) analyser; (**4**) External generator (photo taken by S. Chinchón-Payá).

For the on-site inspection setup, depicted in Figure 2, the hXRF analyser was connected to a laptop computer and both were connected to an external generator. The entire suite fit in the boot of a passenger car. The energy furnished by the batteries in the devices was suffice for spot readings, and therefore there was no need for a generator.

## 3. Results and Discussion

### 3.1. Laboratory Calibration

**Figure 3:**
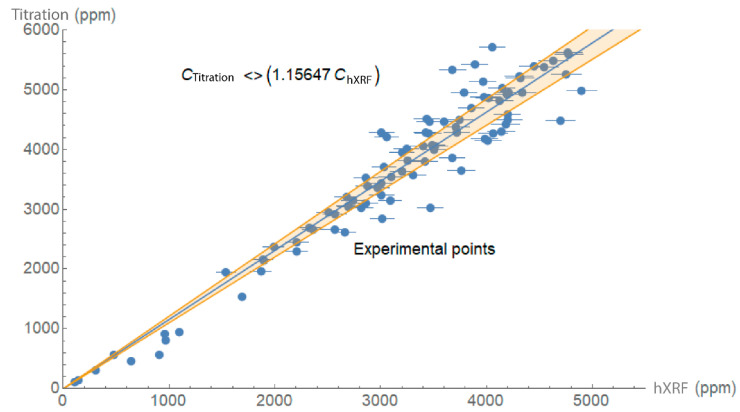
Chloride ions found by titration vs. chloride ions quantified by hXRF (correlation coefficient, R^2^ = 0.92, 90.0% confidence interval).

The end-point potentiometric quantification findings for chloride ions [17] are compared to the hXRF readings for the 91 mortar and concrete samples, shown in Figure 3. The graph attests to the good correlation between the two analytical techniques (R^2^ = 0.92) at a 90.0% confidence interval. Taking the end-point potentiometric value as an accurate measure, the calibration factor for hXRF was found to be 1.16. In other words, the chloride concentration in the structure was equal to the instrumental reading multiplied times 1.16.

### 3.2. On-Site Use of the hXRF Analyser

The inspection findings for the concrete structure studied are discussed below.

As noted earlier, the handheld instrument readings were expressed as chloride concentration by total sample weight, whereas in the legislation the concentration is expressed as a percentage of cement weight. Consequently, when concrete or mortar dosage is unknown, chloride concentration by cement weight must be calculated from assumed values. Given that the cement content in reinforced concrete for structural members usually ranges from 250 to 350 kg per m^3^ of concrete and, in this case study, a closer estimate would be around 250 to 300 kg/m^3^, the value adopted for the calculations was 275 kg/m^3^. Applying the 0.6% (cement weight) chloride concentration threshold set out in the existing legislation yielded a value of 900 ppm as the critical chloride concentration by sample weight.

**Figure 4:**
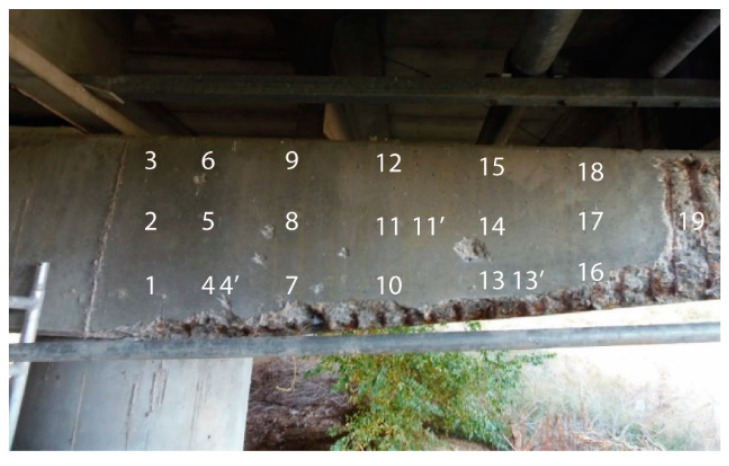
Points measured on the surface of the girder analysed (photo taken by S. Chinchón-Payá).

The corrosion potential and resistivity values data needed to map areas of the element with different degrees of corrosion are given in Table 3 along with the respective corrosion current densities for the points analysed. The table also lists the chloride concentration in the samples analysed and the equivalent in percentage by concrete in cement weight, assuming a cement content of 325 kg per m^3^ of concrete. The points analysed on the girder are marked on the photograph in Figure 4. 

The corrosion potential and the resistivity are used as preliminary measures to delimit possible areas affected by corrosion, due to its ease and speed in their implementation. They are qualitative measurements that locate areas with a high probability of corrosion (19 points from Table 3). These areas are further evaluated by obtaining the concentration of chlorides and the intensity of corrosion that quantifies the corrosion level in areas where no external damage is seen.

Corrosion potential measurements are normally interpreted in terms of the risk involved, as defined by criteria set out in ASTM standard C-876-91 or Spanish standard UNE-112083:2010 (Table 4). The values shown for each level of corrosion were put forward on the grounds of on-site measurements by [5,37,38].

**Figure 5:**
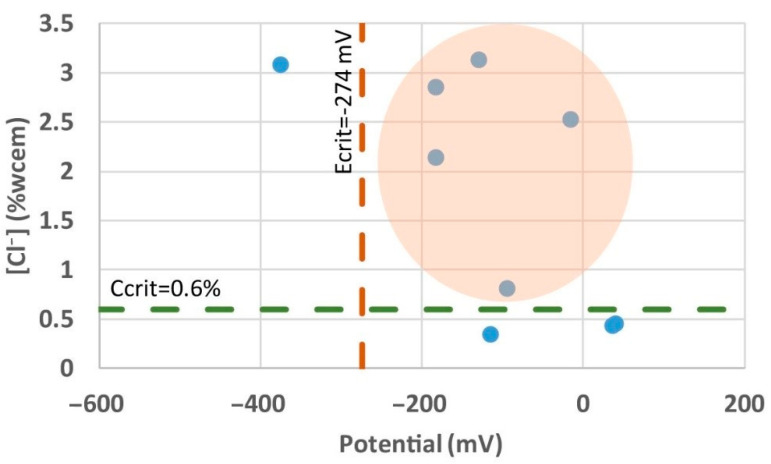
Chloride concentration vs. corrosion potential.

Figure 5 shows the graphs of chloride concentration by cement weight against corrosion potential with Cu/CuSO_4_ as the reference electrode. The dotted lines indicate the critical chloride concentration (Ccrit = 0.6%) and critical corrosion potential (Ecrit = −274 mV). The safe area lies between the values <Ccrit and >Ecrit.

The findings revealed the existence of points (in the shaded area in Figure 5) that, while not activated in terms of corrosion potential (value > Ecrit), exhibited a chloride concentration higher than Ccrit. Although the corrosion potential was <10%, the respective areas were potentially at risk, since the presence of very high chloride concentrations might induce corrosion, sooner or later, prompting reinforcement deterioration.

Corrosion current density, also measured in the area studied, revealed activation at many but not all of the points at issue (*I_corr_* > 0.1 µA/cm^2^). The discrepancy may be attributable to the prevalence of high resistivity over corrosion current density [39,40]. Other effects such as the appearance of macrocells may also be involved [41].

An important consideration, in this context, is that when the areas repaired are adjacent to areas with high chloride content, the likelihood of activation in the latter rises steeply, and with it the risk of drastically reducing repair durability. In situ analyses of the chloride content in conjunction with electrochemical measurements, therefore, contribute to more objective decision making with respect to the area in need of repair.

**Figure 6:**
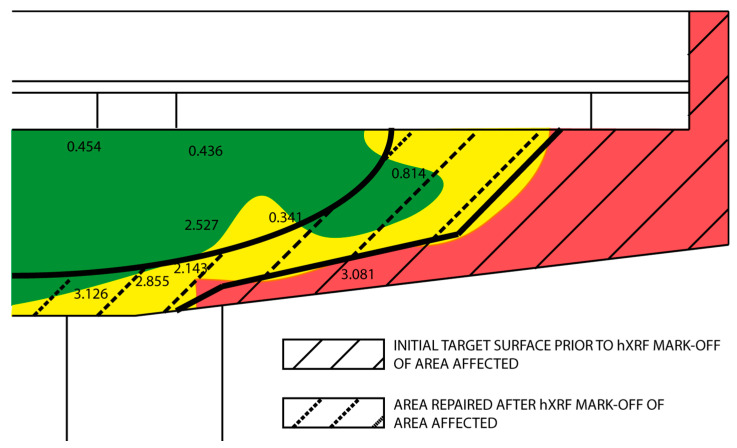
Electrochemical mapping (shading) and chloride concentrations in wt % cement (numbers). Initial (solid black lines) and final areas (dotted black lines) targeted for repair are also shown in straight and dotted lines, respectively.

The corrosion probability diagram, in Figure 6, was derived from potential corrosion and resistivity mapping, and shows the areas of low (green), intermediate (yellow), and high (red) likelihood of corrosion [34,35] on the girder analysed. Figure 6 also includes the chloride concentration values on the grounds of which the area marked off for rehabilitation was enlarged. The initial target and the area actually rehabilitated and repaired on the grounds of the information resulting from this study are shown in Figure 6.

**Figure 7:**
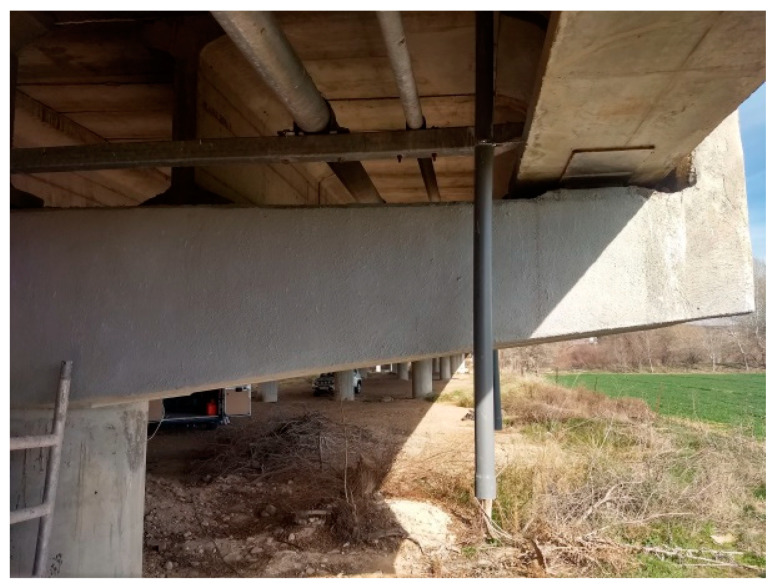
Photograph of concrete member 2 weeks after repair (photo taken by RETINEO).

Figure 7 shows the condition of the surface a few days after repair. The distinction between the areas rehabilitated and the original concrete is not visible because the entire surface was ultimately rendered with mortar. The downpipe visible in the photograph was installed to drain rainwater off the bridge to divert runoff away from the repaired surface.

## 4. Conclusions

The most prominent conclusions drawn from the present study are listed as follows:

- Chloride ions were quantified with a handheld X-ray fluorescence analyser in a large number of mortar and concrete prepared with OPC samples with differing Cl^-^ content. A comparison of the findings to the data delivered by another standardised analytical technique showed the readings to be highly accurate and the precision of the two techniques to be similar. The correction factor calculated for the analyser was 1.16.
- On the grounds of the 0.6% chloride threshold (by cement weight) laid down in the legislation applied, a value of 900 ppm was established as a conservative threshold above which chloride ion-mediated corrosion may be deemed to be underway or potentially underway. The methodology described is equally applicable for use with other standards or codes with different thresholds for calculating the respective chloride concentration threshold.
- This study discusses the use of a handheld X-ray fluorescence analyser to support analysis in repairs, where the primary cause of deterioration is reinforcement steel oxidation due to the presence of a high chloride concentration. Such instruments furnish speedy and accurate on-site information on ion concentration in concrete that can, then, be applied to more accurately define the areas in need repair.

## Figures and Tables

**Table 1:** Corrosion risk assessed in terms of resistivity (ρ).

Resistivity (ρ)	Corrosion Risk
>1000–2000 Ω·m	Steel activity/passivity indistinguishable, low corrosion risk, irrespective of chloride content or degree of carbonation
>500–1000 Ω·m	Low
>100–500 Ω·m	Moderate to high in concretes exposed to carbonation or chloride attack
<100 Ω·m	Process not governed by resistivity, highest possible risk of concrete corrosion

**Table 2:** Corrosion risk assessed in terms of corrosion current density (*i_corr_*).

Corrosion Current Density (*i_corr_*) (μA/cm^2^)	Corrosion Risk
<0.1	Negligible
0.1 a 0.5	Low
0.5 a 1	Moderate
>1	High

**Table 3:** Corrosion parameters, hXRF chloride quantification, and per cent of chloride ions by cement weight.

Point	Corrosion Potential and Resistivity Mapping		Corrosion Rate	Chloride Concentration	
	E_corr_ (mV_Cu/CuSO4_) (±10)	Resistivity (Ω·m) (±10)	*I_corr_* (µA/cm^2^) (±0.002)	[Cl] (ppm) (±10)	[Cl] (wt % cem.) (±0.001)
1	−129	8090	0.013	3738	3.126
2	−25.8	>9990			
3	39.6	>9990		543	0.454
4	−182	3320		3414	2.855
4	−182	3320	0.044	2562	2.143
5	−15.6	>9990		3021	2.527
6	36.1	>9990		521	0.436
7	−415	8480			
8	−115	>9990		408	0.341
9	8.9	>9990			
10	−375	2280	0.493	3684	3.081
11	−94	3810			
11	−94	3810	0.801	973	0.814
12	−248	2970			
13	−277	3840			
13	−277	3840	0.393		
14	−248	280			
15	−248	7990			
16	−319	301			
17	−393	350	0.865		
18	−266	380			
19	−265	290			

**Table 4:** Corrosion risk assessed in terms of corrosion potential.

Potential (mV)	Ccorrosion Risk	Saturated Calomel Elec−Trode (SCE)	Ag/AgCl Electrode (KCl_3M_−Saturated)	Cu/CuSO_4_ Electrode (Cu^2+^−Saturated)
Passivity	10%	>−200	>−157	>−274
Intermediate status	50%	−200 to −350	−157 to −307	−274 to −424
High risk	90%	<−350	<−307	<−424

## Data Availability

The data presented in this study are available on request from the corresponding author. The data are not publicly available due to they belong to RETINEO.
